# Evaluation of clinical and imaging features for differentiating rhabdomyosarcoma from neuroblastoma in pediatric soft tissue

**DOI:** 10.3389/fonc.2024.1289532

**Published:** 2024-02-09

**Authors:** Jing Sheng, Tingting Li, Huafeng Xu, Rong Xu, Xuemei Cai, Huanhuan Zhang, Qiongqiong Ji, Xiuhua Duan, Weiwei Xia, Xiujun Yang

**Affiliations:** Department of Radiology, Shanghai Children’s Hospital, School of Medicine, Shanghai Jiao Tong University, Shanghai, China

**Keywords:** rhabdomyosarcoma, neuroblastoma, computed tomography, magnetic resonance imaging, differentiation

## Abstract

**Background:**

In this study, we developed a nomogram predictive model based on clinical, CT, and MRI parameters to differentiate soft tissue rhabdomyosarcoma (RMS) from neuroblastoma (NB) in children preoperatively.

**Materials and methods:**

A total of 103 children with RMS (n=37) and NB (n=66) were enrolled in the study from December 2012 to July 2023. The clinical and imaging data (assessed by two experienced radiologists) were analyzed using univariate analysis, and significant factors were further analyzed by multivariable logistic regression using the forward LR method to develop the clinical model, radiological model, and integrated nomogram model, respectively. The diagnostic performances, goodness of fit, and clinical utility of the integrated nomogram model were assessed using the area under the curve (AUC) of the receiver operator characteristics curve (ROC) with a 95% confidence interval (95% CI), calibration curve, and decision curve analysis (DCA) curves, respectively. Diagnostic efficacy between the model and radiologists’ interpretations was examined.

**Results:**

The median age at diagnosis in the RMS group was significantly older than the NB group (36.0 months vs. 14.5 months; *P*=0.003); the fever rates in RMS patients were significantly lower than in patients with NB (0.0% vs.16.7%; *P*=0.022), and the incidence of palpable mass was higher in patients with RMS compared with the NB patients (89.2% vs. 34.8%; *P*<0.001). Compare NB on image features: RMS occurred more frequently in the head and neck and displayed homogeneous density on non-enhanced CT than NB (48.6% vs. 9.1%; 35.3% vs. 13.8%, respectively; all *P*<0.05), and the occurrence of characteristics such as calcification, encasing vessels, and intraspinal tumor extension was significantly less frequent in RMS children compared to children with NB (18.9% vs. 84.8%; 13.5% vs. 34.8%; 2.7% vs. 50.0%, respectively; all *P <*0.05). Two, three, and four features were identified as independent parameters by multivariate logistic regression analysis to develop the clinical, radiological, and integrated nomogram models, respectively. The AUC value (0.962), calibration curve, and DCA showed that the integrated nomogram model may provide better diagnostic performance, good agreement, and greater clinical net benefits than the clinical model, radiological model, and radiologists’ subjective diagnosis.

**Conclusion:**

The clinical and imaging features-based nomogram has potential for helping radiologists distinguish between pediatric soft tissue RMS and NB patients preoperatively, and reduce unnecessary interventions.

## Introduction

Rhabdomyosarcoma (RMS) is the most prevalent aggressive malignant soft tissue sarcoma in children, originating from primitive mesenchymal stem cells and arising in various anatomic sites throughout the body (1). Besides, RMS is the third most prevalent pediatric extracranial solid tumor, following neuroblastoma (NB) and Wilms tumor, with an incidence of 0.44 cases per 100,000 individuals per year (2, 3). NB is a malignancy develops from immature nerve cells located in several areas of the body, including the adrenal glands, neck, chest, abdomen, and spine (4). The prevalence of NB is estimated to be 10.2 cases per million children aged 15 and below, constituting almost 15% of all pediatric cancer-related fatalities (5–7). Previous studies indicated that RMS and NB, especially in soft tissue, share comparable anatomical locations, aggressive tumor behavior, immunohistochemical features, and clinical presentation characteristics of childhood solid tumors (8, 9), making preoperative stage differential diagnosis challenging (10).

Despite these similarities, the management strategies and prognoses for RMS and NB differ. Children diagnosed with RMS typically have surgery, while NB treatment varies by risk category and may include chemotherapy, surgery, radiation, stem cell transplant, immunotherapy, and retinoid therapy (3, 11). In addition, the 5-year relative survival rate for low-risk and localized RMS in children exceeds 80% with surgery, radiation therapy, and chemotherapy. However, in high-risk RMS or metastatic cases, the rate drops below 30%. For neuroblastoma (NB), the 5-year relative survival rates are over 95%, 90-95%, and around 50% for low-, intermediate-, and high-risk cases, respectively (12). Currently, post-operative histology is the only reference standard for the differential diagnosis of RMS versus NB. However, this method is invasive, tends to cause patients discomfort, and risk of complications. Thus, it is crucial to develop non-invasive and effective methods for distinguishing the two tumors, as this could potentially eliminate the need for tissue biopsies in risky anatomical sites.

Science defining the clinical characteristics of RMS alone is frequently insufficient to distinguish it from NB; imaging techniques for its identification become necessary (13). Computed tomography (CT) and magnetic resonance imaging (MRI), being widely employed imaging modalities, serve a critical function in providing indispensable information to facilitate precise diagnosis, staging, treatment strategizing, and follow-up in patients affected with RMS and NB. Although several morphological CT and MRI characteristics, including soft tissue density, adjacent bone destruction (approximately 20% of cases), heterogeneous enhancement, and others, have been associated with RMS, there are still numerous cases in which these signs are absent or not apparent. Furthermore, imaging findings that can differentiate between RMS and NB have not yet been established (14–16).

Therefore, the purpose of the present study was to develop an integrated nomogram model incorporating clinical and imaging variables to distinguish preoperatively between RMS and NB for precision therapy. Subjects enrolled in this study mainly concentrated on patients with RMS and NB in the context of pediatric soft tissue tumors, considering their propensity to develop extracranially and RMS’s preference for soft tissue involvement.

## Materials and methods

### Patients

**Figure 1:**
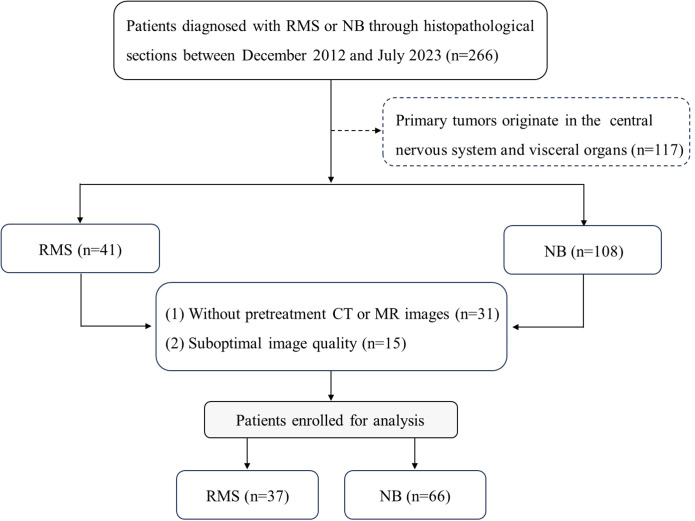
Patient screening flowchart.

For this retrospective study, approval from the Ethical Committee of the Children’s Hospital of Shanghai/Shanghai Children’s Hospital, Shanghai Jiao tong University (Approval number: 2023R073-E01) was obtained, and the requirement for informed consent was waived. One hundred and three consecutive patients with histopathologically confirmed RMS (n=37) or NB (n=66) were included between December 2012 and July 2023. The following was a prerequisite for inclusion: (1) histopathological confirmation of RMS or NB; (2) CT or MRI scans were performed prior to treatment; (3) availability of an adequate assessment of clinical features. The factors for exclusion are listed below: (1) initial tumors originate in the visceral organs and the central nervous system; (2) image artifacts and overlaps lead to poor or damaged image quality and cannot be diagnosed; (3) the presence of other tumors. As shown in **Figure 1**.

### Clinical data

The clinical characteristics of all eligible patients, including age, gender, and associated symptoms at diagnosis (**Table 1**), were retrospectively gathered from the Hospital Information System (HIS) of Shanghai Children’s Hospital. Two radiologists with 10 to 15 years of experience reviewed these data for each patient and recorded them.

**Table 1:** Clinical characteristics of RMS and NB patients in the present study.

Characteristics	RMS (n=37)	NB (n =66)	*P* Value
Age (months), median (IQR)	36.0 (17.0-68.0)	14.50 (6.0-40.0)	0.003
Gender, n (%)			
Male	26 (70.3)	38 (57.6)	0.203
Female	11 (29.7)	28 (42.4)	
Symptoms, n (%)			
Fever	0 (0.0)	11 (16.7)	0.022
Pain	11 (29.7)	11 (16.7)	0.121
Palpable mass	33 (89.2)	23 (34.8)	<0.001

IQR, interquartile range; RMS, rhabdomyosarcoma; NB, neuroblastoma.

### Protocols of radiological examinations

Preoperative CT examinations (n=99) were conducted using multidetector CT machines (LightSpeed 64, GE Healthcare, USA; Aquilion 64, TOSHIBA, Japan). The scans were mainly performed using the following parameters: 0.984 pitch, 100-120 kV, automatic tube current modulation, interval of 5 mm, and a layer thickness of 5 mm. What’s more, coronal and sagittal reformatted images were obtained based on axial images with 3 mm section thickness. Intravenous administration of nonionic contrast media (1-1.5 mL/kg Iohexol, 350 mg/ml, Schering, Germany) was injected at a rate of 2-3 ml/s using an automatic power injector (OptiVantage DH; Mallinckrodt, St. Louis, Mo) into a 22- or 24-gauge intravenous cannula, without normal saline bolus administration.

Preoperative MRI examinations (n=36) were performed using a 3.0 T scanner (Ingenia, Philips Medical Systems, Best, the Netherlands). To achieve optimal resolution, coils were chosen according to the examined body part. The standardized MRI protocol mainly included T1-weighted images (T1WI; repetition time (TR)/echo time (TE): 615/18 ms; section thickness/gap: 4/0.4 mm; matrix: 300×200 mm; field of view: 24×20 cm), T2-weighted images (T2WI; TR/TE: 2800/85 ms; section thickness/gap: 4/0.4 mm; matrix: 368×210 mm; field of view: 24×20 cm), fat-suppressed T2-weighted images (Fs-T2WI), and contrast-enhanced (CE) T1WI (CE T1WI; TR/TE: 580/15 ms; section thickness/gap: 4/0.4 mm; matrix: 300×200 mm; field of view: 24×20 cm). CE T1WI were obtained after 0.2 ml/kg Gd-DTPA (3 ml/s) was injected intravenously. Diffusion-weighted images (DWI) were obtained by a single-shot echo with b-values of 800 s/mm2 for the following parameters: TR/TE, 2255/65 ms; section thickness/gap, 4/0.4 mm; matrix, 88 x 100 mm; field of view, 22×20 cm. We applied the diffusion gradients in three orthogonal directions (x, y, and z). Apparent diffusion coefficient (ADC) maps were calculated from the DWI data.

### Image analysis

Two board-certified radiologists with 10 and 15 years of experience in soft tissue image interpretation independently reviewed preoperative CT and MR images using the picture archiving and communication systems (PACS). Both readers were blinded to the pathological diagnosis as well as any clinical or laboratory information; however, they were aware that those images had been obtained from patients with soft tissue tumors. After their first independent reading, the two radiologists reviewed and discussed any differences in their interpretations of subjective parameters in order to come to a consensus.

The following signs were noted and documented: (1) the predominant location of tumors; (2) the number of lesions (single or multiple); (3) tumor shape (round, lobulated, or irregular); (4) size (maximum tumor diameter); (5) border (well-defined or ill-defined); (6) calcification, cystic degeneration, hemorrhage, encasing vessel, midline crossing (present or absent); (7) density/signal intensity (hypo-, iso-, or hyperdense/intense relative to the same level); (8) diffusion restriction, refers to high signal intensity on high b-value DWI (b = 800 s/mm^2^) with a low value on the corresponding ADC maps; (9) homogeneity, and enhancement characteristics. Other imaging features, such as adjacent bone destruction, intraspinal tumor extension, and enlarged lymph nodes, were also examined. The maximum tumor diameter was determined in any axial, coronal, or sagittal plane on both CT and MRI. Criteria for vascular encasement were tumor contact of at least 50% of the vessel’s circumference (17).

### Integrated nomogram construction and evaluation

In order to further construct a better model for identifying RMS from NB, we developed an integrated nomogram constructed by multiple logistic regression, incorporating statistically significant clinical factors and imaging features. The area under the curve (AUC) of the receiver operator characteristics curve (ROC) with a 95% confidence interval (95% CI) has been developed to measure the predictive performance, and integrated discrimination improvement (IDI) was calculated to evaluate the performance between the three models. The goodness of fit for the final multiple logistic regression model was further assessed using the Hosmer-Lemeshow test, and the calibration plot was drawn. Finally, we plotted decision curve analysis (DCA) curves for the three models to display the overall net benefit performance of the integrated nomogram model.

### Radiologist’s diagnosis

Three radiologists, with various amounts of experience in diagnosing children’s cancer (senior: 10 years; middle-aged: 5 years; junior: 1 year), independently interpreted the patients involved in the study without any prior knowledge of the final pathological results. Evaluated and compared the diagnostic efficacy of each radiologist, using the pathological data as the gold standard. The diagnostic efficacy was evaluated using ROC curves, which considered AUC, accuracy, sensitivity, and specificity variables.

### Statistical analysis

All statistical analyses in the present study were performed using SPSS version 25.0 (SPSS, Chicago, IL, USA) and R version 4.3.1. Continuous variables were presented as mean and standard deviation (SD) or median with interquartile range (IQR), and were compared using an independent-samples *t*-test or the Mann-Whitney U test. Categorical variables were shown as numbers and percentages, and were analyzed by the Fisher’s exact test or the chi-square test. Factors with statistical significance by univariate analysis were further analyzed by multivariable logistic regression using the forward LR method, and the clinical model, radiological model, and integrated nomogram model were established, respectively. Inter-reader reliability was measured with Cohen’s kappa statistic. The DeLong test was used to compare the difference of AUCs between three kinds of models or between three radiologists’ subjective diagnosis. A *P* value of <0.05 was considered statistically significant for all the statistical tests.

## Results

### Patients and clinical characteristics

The study included a total of 103 eligible patients, consisting of 37 (35.9%) children diagnosed with RMS and 66 (64.1%) children diagnosed with NB. The median age at diagnosis in the RMS group was 36.0 months, which was significantly higher than the 14.5 months of the patients in the NB group (*P*=0.003). In terms of the early clinical signs, the incidence of fever in patients with RMS was considerably lower compared to patients with NB (0.0% vs. 16.7%; *P*=0.022). Additionally, the RMS group had significantly greater rates of palpable mass compared to the NB group (89.2% vs. 34.8%; *P*<0.001). No significant differences were identified in gender or presentation of pain between groups (all *P >*0.05). As listed in **Table 1**.

### Radiological features

**Figure 2:**
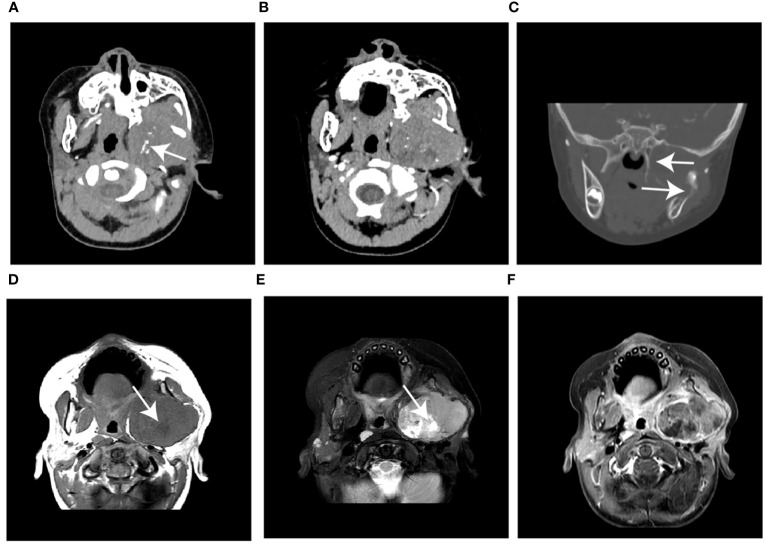
Case 1, a 21-month-old boy with RMS. **(A)** The unenhanced CT image shows a huge, slightly hypodense mass in the left infratemporal fossa with speckled calcification (arrow). **(B)** The mass shows heterogeneous enhancement without encasing vessel signs on contrast-enhanced CT. **(C)** The coronal reconstructed CT image shows extensive erosion of adjacent bones (arrow). **(D)** Axial T1WI and **(E)** T2WI show the mass with heterogeneous signals intensity with cystic lesions (arrow). **(F)** The gadolinium-enhanced T1WI shows a hyperintense solid mass compared with the adjacent muscle layer. WI, weighted image.

**Figure 3:**
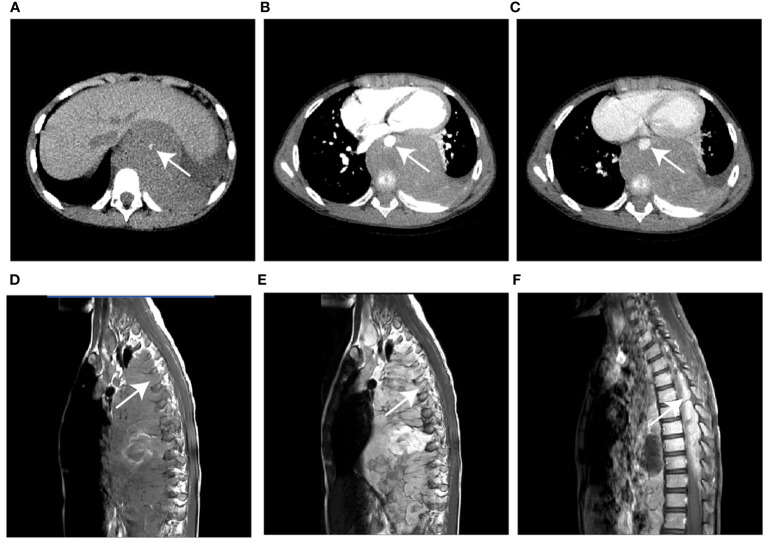
Case 2, a 76-month-old male with NB. **(A)** The unenhanced CT image shows a hypodense mass in the left posterior mediastinum with punctate calcification (arrow) **(B, C)** The mass shows heterogeneous enhancement and encases the aorta (arrow) on contrast-enhanced CT images. **(D)** Sagittal T1WI and **(E)** T2WI show the soft tissue mass extended into the spinal canal via the intervertebral foramen (arrow). **(F)** The gadolinium-enhanced T1WI shows the intraspinal extent of the tumor (arrow). WI, weighted image.

The initial RMS locations were the head and neck (48.6%), and the trunk and extremities (51.4%), which differed significantly from the children in the NB group 9.1%, 90.9%, respectively; *P*<0.001). On CT images, the RMS tumors appeared homogeneity (35.3%), which was higher than the NB (13.8%), and the difference was statistic (*P=*0.013) (**Figure 2**). In contrast, the rates of calcification, encasing vessels, and intraspinal tumor extension were significantly lower in the RMS group compared to the NB group (18.9% vs. 84.8%; 13.5% vs. 34.8%; 2.7% vs. 50.0%, respectively; all *P <*0.05) (**Figure 3**). Concerning other characteristics such as the lesional number, shape, size, border, cystic lesions, hemorrhage, midline crossing, and adjacent bony destruction, there were no statistically significant differences observed between the groups (all *P*>0.05). In terms of MRI observations, in 17 cases of RMS and 19 cases of NB, tumors exhibited hypointense or isointense signals (88.2% vs. 73.7%), homogeneous signals (29.4% vs. 21.1%) on T1WI, hyperintensity signals (94.1% vs. 100.0%), heterogeneous signals (100.0% vs. 94.7%) on T2WI, respectively (all *P*>0.05). In addition, the incidence of diffusion restriction, homogeneous enhancement, and enlarged lymph node of the lesions in the RMS group was not significantly different compared with the NB group (all *P*>0.05) (**Table 2**). Regarding inter-reader reproducibility, the Kappa values for all findings exceeded 0.8, thereby proving its substantial reproducibility and applicability.

**Table 2:** Comparison of imaging features between RMS and NB.

Characteristics	RMS (n=37)	NB (n =66)	*P* Value
Location, n (%)			<0.001
Head and neck	18 (48.6)	6 (9.1)	
Trunk and extremities	19 (51.4)	60 (90.9)	
Number of lesions, n (%)			0.082
Single	34 (91.9)	66 (100.0%)	
Multiple	3 (8.1)	0 (0.0)	
Shape of the tumor, n (%)			0.964
Round	13 (35.1)	24 (36.4)	
Lobulated	4 (10.8)	8 (12.1)	
Irregular	20 (54.1)	34 (51.5)	
Size (mm), mean ± SD	7.54 ± 5.98	6.87 ± 3.35	0.326
Tumor border, n (%)			0.829
Ill-defined	21 (56.8)	36 (54.5)	
Well-defined	16 (43.2)	30 (45.5)	
CT attenuation, n (%)^*^			
Hypodense or iso-dense	31/34 (91.2)	61/65 (93.8)	0.937
Homogeneous	12/34 (35.3)	9/65 (13.8)	0.013
Calcification, n (%)	7 (18.9)	56 (84.8)	<0.001
Cystic degeneration, n (%)	16 (43.2)	17 (25.8)	0.068
Encasing vessels, n (%)	5 (13.5)	23 (34.8)	0.020
Hemorrhage, n (%)	3 (8.1)	4 (6.1)	1.000
Midline crossing, n (%)	17 (45.9)	43 (65.2)	0.058
Intraspinal tumor extension, n (%)	1 (2.7)	33 (50.0)	<0.001
Bony destruction, n (%)	10 (27.0)	11 (16.7)	0.211
T1WI, n (%)^†^			
Hypointense or isointense	15/17 (88.2)	14/19 (73.7)	0.408
Homogeneous	5/17 (29.4)	4/19 (21.1)	0. 706
T2WI, n (%)^†^			
Hyperintense	16/17 (94.1)	19/19 (100.0)	0.472
Heterogeneous	17/17 (100.0)	18/19 (94.7)	1.000
Diffusion restriction, n (%))^†^	12/13 (92.3)	17/17 (100.0)	0.433
Heterogeneous enhancement, n (%)^‡^	37/37 (100.0)	60/64 (93.8)	0.307
Swollen lymph nodes, n (%)	14 (37.8)	22 (33.3)	0.646

CT, computed tomography; T1WI, T1-weighted image; T2WI, T2-weighted image; SD, standard deviation; RMS, rhabdomyosarcoma; NB, neuroblastoma.

^*^ 99 patients underwent CT scans.

^†^ 30 patients underwent DWI, while 36 patients underwent T1WI and T2WI.

^‡^ 101 patients underwent contrast-enhanced CT or MRI scans.

### Construction of the models

To predict the probability of discrimination between RMS and NB tumors, all factors with a *P*-value < 0.05 in the univariate analysis were incorporated into the forward stepwise multivariate logistic regression analysis. Based on independent factors identified by logistic regression analysis, the results revealed that age (OR, 1.03; 95% CI,1.01-1.05; *P*<0.001), and palpable mass (OR, 35.12; 95% CI, 7.08-174.29; *P*<0.001) were independent variables to differentiate RMS and NB in the clinical model. Location (OR, 0.18; 95% CI, 0.04-0.73; *P*=0.016), calcification (OR, 0.06; 95% CI, 0.02-0.21; *P*<0.001), and intraspinal tumor extension (OR, 0.06; 95% CI, 0.01-0.57; *P*=0.015) were independent factors in the radiological model. Following the construction of a merged model, the factor of location ceased to be regarded as an independent predictor for distinguishing between the two tumors. Therefore, only four variables, including age (OR, 1.04; 95% CI, 1.01-1.08; *P* = 0.021), palpable mass (OR, 20.92; 95% CI, 3.25-134.47; *P*=0.001), calcification (OR, 0.05; 95% CI, 0.01-0.24; *P*<0.001), and intraspinal tumor extension (OR, 0.04; 95% CI, 0.00-0.79; *P*=0.034), were included in the integrated nomogram model (**Table 3**).

**Table 3:** Multivariable logistic analysis of predictors of RMS with confidence interval.

Characteristics	Clinical model		Radiological model		Integrated nomogram model	
	OR (95%CI)	*P* Value	OR (95%CI)	*P* Value	OR (95%CI)	*P* Value
Age (months)	1.03 (1.01-1.05)	*<*0.001	–	–	1.04 (1.01-1.08)	0.021
Fever	NS	–	–	–	NS	–
Palpable mass	35.12 (7.08-174.29)	*<*0.001	–	–	20.92 (3.25-134.47)	0.001
Location	–	–	0.18 (0.04-0.73)	0.016	NS	–
Homogeneous density	–	–	NS	–	NS	–
Calcification	–	–	0.06 (0.02-0.21)	*<*0.001	0.05 (0.01-0.24)	*<*0.001
Encase vessels	–	–	NS	–	NS	–
Intraspinal tumor extension	–	–	0.06 (0.01-0.57)	0.015	0.04 (0.00-0.79)	0.034

OR, odds ratio; CI, confidence interval; NS, not significant.

### Comparison between different models

**Figure 4:**
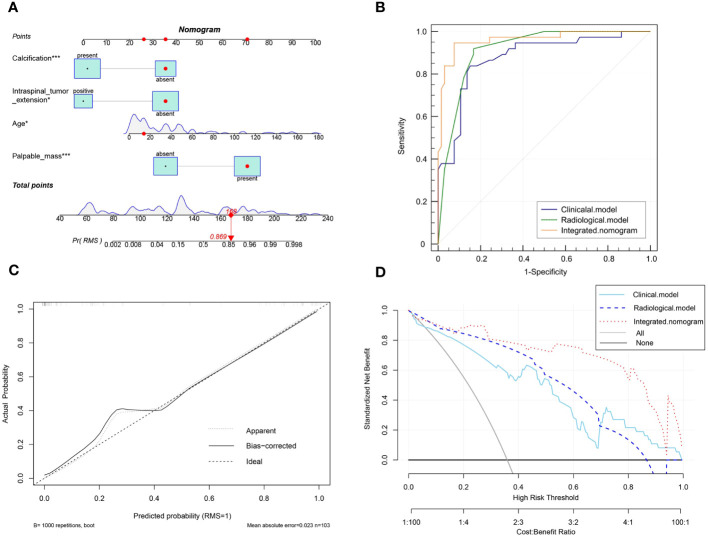
**(A)** The visual nomogram in the integrated model. **(B)** The receiver operator characteristics (ROC) curves of the three different models. **(C)** Calibration curves of the integrated nomogram model. **(D)** The decision curve analysis (DCA) for the clinical model, radiological model, and integrated nomogram model.

A nomogram was built to visualize and estimate the probability of the RMS based on these predictors in the combined model (**Figure 4A**). The data indicated that the C index was 0.961, suggesting that the nomogram model possessed good differentiating capability. The ROC curve threshold analysis calculated the AUC value, accuracy, sensitivity, and specificity for each model (**Table 4**). **Figure 4B** demonstrates that the integrated nomogram model exhibited the highest AUC value of 0.962 (95% CI, 0.905-0.990), followed by the radiological model [0.915 (95% CI, 0.843-0.961)] and the clinical model [0.878 (0.799-0.934)], and achieved the best diagnostic performance than the clinical model (IDI=0.291, 95% CI, 0.205-0.377; *P*< 0.001) and the radiological model (IDI=0.162, 95% CI, 0.071-0.253; *P*< 0.001). **Figure 4C** reveals a strong concordance between the predicted values and the observed values, as evidenced by a *P*=0.125 in the Hosmer-Lemeshow test. The DCA curves for the three models are displayed in **Figure 4D**, indicating that the integrated nomogram model provides greater benefits to patients compared to both the treat-no-patient schemes and the treat-all-patient regimens. Moreover, the DCA curves found that the integrated nomogram model exhibited superior net benefit compared to both the clinical and radiological models in accurately diagnosing individuals with RMS.

**Table 4:** Diagnostic performances of the three models.

Models	AUC (95% CI)	Accuracy (95% CI)	Sensitivity (95% CI)	Specificity (95% CI)
Clinical model	0.878 (0.799-0.934)	0.845 (0.842-0.847)	0.838 (0.680-0.938)	0.849 (0.739-0.925)
Radiological model	0.915 (0.843-0.961)	0.864 (0.862-0.866)	0.919 (0.781-0.983)	0.833 (0.721-0.914)
Integrated nomogram model	0.962 (0.905-0.990)	0.932 (0.931-0.933)	0.946 (0.818-0.993)	0.924 (0.832-0.975)

AUC, area under the curve; CI, confidence interval.

### Comparative diagnostic efficacy between radiologists and the model

**Figure 5:**
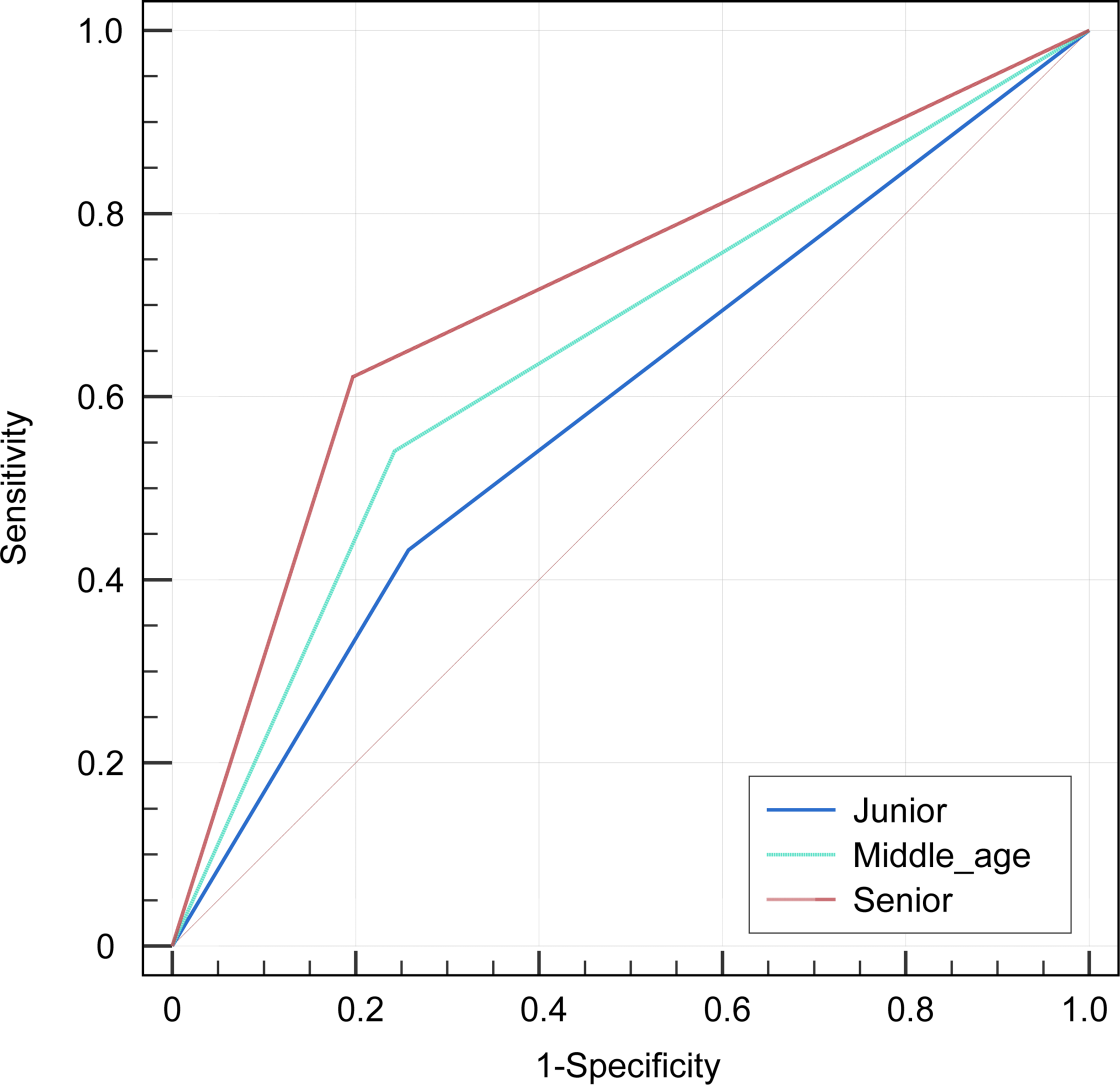
The receiver operator characteristics (ROC) curves indicate the subjective diagnosis performance of a junior (blue line), middle-aged (green line), and senior (red line) radiologist. The calculated values for the area under the curve (AUC) were 0.587, 0.649, and 0.699, in that order.

Among the three radiologists, the AUC value of 0.699 (95% CI, 0.605-0.792) of the senior radiologist was the highest, which was significantly higher than the values of the middle-aged [0.649 (95% CI, 0.552-0.746), *P*=0.029] and junior ones [0.587 (0.491-0.684), *P*= 0.001]. Refer to **Table 5** and **Figure 5** for the specific results. Nevertheless, the diagnostic efficacy of the senior radiologist was considerably lower, when compared to the integrated nomogram model [0.962 (95% CI, 0.905-0.990), *P*<0.001)].

**Table 5:** The subjective results of three radiologists.

Experience	Diagnosis	Pathological result		Total	AUC (95% CI)	Accuracy (95% CI)	Sensitivity (95% CI)	Specificity (95% CI)
		RMS (n=37)	NB (n =66)	103				
Junior	RMS	16	17	33	0.587 (0.491-0.684)	0.631 (0.627-0.635)	0.432 (0.273-0.592)	0.742 (0.637-0.848)
	NB	21	49	70				
Middle-age	RMS	20	16	36	0.649 (0.552-0.746)	0.680 (0.675-0.684)	0.541 (0.380-0.701)	0.758 (0.654-0.861)
	NB	17	50	67				
Senior	RMS	22	13	35	0.699 (0.605-0.792)	0.728 (0.724-0.731)	0.595 (0.436-0.753)	0.803 (0.707-0.899)
	NB	15	53	68				

RMS, rhabdomyosarcoma; NB, neuroblastoma; AUC, area under the curve; CI, confidence interval.

## Discussion

There have been many efforts to distinguish between RMS and NB to improve diagnostic accuracy and avoid unnecessary intervention (18–20). In the present study, we developed and validated the clinical model, radiological model, and integrated nomogram model, combining clinical findings and imaging features, as a novel and effective complementary method for preoperative identification of children with RMS and NB originated in soft tissue. Furthermore, the ROC, calibration curve, and decision curve were used to assess the discrimination ability, accuracy, and clinical benefit of the model, respectively. All assessment indicators revealed that the integrated nomogram model was superior to the single model and subjective diagnosis in distinguishing RMS from NB patients, and the nomogram model was a potentially effective tool for the need for anti-tumor therapy.

Clinical findings of both RMS and NB may show a wide range of differences in the initial differentiation. Liu et al. (21) retrospectively analyzed the follow-up data of 20 children with RMS and found that the most common clinical symptoms of the RMS children at the first visit were painless soft tissue masses (13/20), with a median age at diagnosis of 48 months. Additionally, pediatric RMS incidence also varies by gender, as male children have a higher incidence of RMS compared to female children (OR, 1.51; 95% CI, 1.27-1.80) (22). On the contrary, symptoms of NB vary with site of presentation and include fever, emesis, fatigue, and abdominal masses that often manifest with constipation and abdominal distention that may be painful (23). Fever is a common symptom and is present in 26.0% of pediatric patients with NB, and NB is commonly diagnosed as cancer in infancy, with the median age at diagnosis being approximately 19 months (24, 25). Our study has shown similar results: when comparing patients with NB, the median incidence age of children with RMS was higher, and the symptoms of mass were more common (all *P*<0.05). Meanwhile, 16.7% of the NB children (11/66) presented fever, compared with none of the RMS patients (*P*=0.022), and no significant correlation was observed between gender and tumor incidence, nor were there any notable changes in other clinical features between the two groups (all *P*>0.05).

Further, the present study evaluated the performance of these radiographic features in discriminating between the two tumors when applied to CT and MRI. Head and neck sites account for close to 50% of RMS cases (26). In a retrospective study, including 10 head and neck RMS patients, conducted by Zhu et al., it was found that eight of the RMS patients appeared on the CT images to be slightly hypodense (2/8) or iso-dense (6/8) with homogeneous enhancement (4/4), and the soft tissue masses had poorly defined borders (9/10), bony destruction (10/10), and multi-cavity growth (7/10), but calcification and hemorrhaging were not found. On T1WI, nine of the nine tumors exhibited iso-intensity, and on T2WI, six tumors exhibited homogeneous hyperintensity with homogeneous enhancement on contrast-enhanced (CE)-T1WI, and the lesion is typically heterogeneously hyperintense to muscle on T2WI owing to hemorrhage or necrosis (27). The imaging results of the RMS in the present study were similar to those of previous studies. Tian et al. (28) reviewed 37 children’s radiographic data with pelvic RMS and reported that the imaging features indicating lower than normal muscle density, necrosis, non-calcification, and non-hemorrhage exhibited high specificity (95% CI, 0.86-0.97), but the sensitivity (95% CI, 0.32-0.40) was relatively low. According to our study, thirty-one RMS cases had hypodense or iso-dense, ill-defined borders (21/34), cystic degeneration (16/34), calcification (7/34), hemorrhaging (3/34), and bony destruction (10/34). Moreover, most masses are hypointense to iso-intense on T1WI (15/17) and hyperintense on T2WI (16/17) while enhancing heterogeneously with contrast material in all cases. For NB patients reported in previous studies, the images suggested poorly marginated, heterogeneous masses, and one of the key defining features is the presence of calcification seen in 80-90% of the cases. Furthermore, NB tend to demonstrate extension across the midline and into adjacent body cavities, and they tend to encase and displace structures rather than invade them, such as encasing vessels, which may lead to compression (29). MRI should now be the preferred imaging modality for all primary NB tumors, as it is superior to CT in assessing metastatic bone marrow disease, chest wall invasion, and spinal canal involvement and can readily assess the organ of origin as well as disease extent (30). On MRI at diagnosis, NB typically return low signal on T1W sequences and high signal on T2 sequences. In addition to calcification and hemorrhage, areas of variable contrast enhancement and restricted diffusion on DWI can be observed in malignant lesions (14, 15). Consistently, from the results of the current study, NB tumors had poorly marginated (36/66), heterogeneous mass (56/65), calcification (56/66), encasing vessels (23/66), extension across the midline (43/66), and intraspinal tumor extension (33/66), hypointense or isointense signal on T1 sequences (14/19), hyperintense signal on T2 sequences (19/19), and diffusion restriction (17/17). However, there were few hemorrhages (4/66) observed (**Figure 3**). The outcome of comparing the CT and MRI features of the two tumors revealed that while the original locations of RMS tumors in the head and neck were more common than NB (48.6% vs. 9.1%, *P*<0.001), the imaging features of RMS lesions in heterogeneous intensity, calcification, encased vessels, and intraspinal tumor extension were less frequent than NB (all *P*<0.05). Therefore, to differentiate between RMS and NB based on tumor location, density, and calcification, prioritize CT. To further identify the intricate details of soft tissue, like the presence of encased vessels and intraspinal tumor extension, an MRI should be used. Further, it is critical to differentiate other possible soft tissue masses that can be encountered in children with NB and RMS, such as musculoskeletal soft tissue infections (pyomyositis), soft tissue lymphoma (non-Hodgkin lymphoma), and others (lipomas). The imaging diagnosis of pyomyositis differs from RMS and NB due to the presence of focal muscle involvement and well-defined fluid accumulation on MRI (31, 32). These findings may indicate inflammatory changes and abscess formation. In contrast, non-Hodgkin lymphoma is characterized by solid-enhancing tumors in lymph nodes or extranodal locations and liver or spleen involvement (33). Lipomas always have obvious boundaries, no contrast enhancement, and fat saturation sequence signal suppression (34). Thus, they can be recognized from RMS and NB in children.

To obtain an appropriate model able to distinguish between RMS and NB, we developed clinical, radiological, and integrated nomogram models by incorporating significant variables (*P*<0.05) from the univariate analysis into forward stepwise multivariate logistic regression. Although previous studies did not report the typical clinical symptoms and features differences between the two tumors (35), the clinical model demonstrated good results in discriminating pediatric RMS and NB, with an AUC value of 0.878 (95% CI, 0.799-0.934), accuracy of 0.845 (95% CI, 0.842-0.847), sensitivity of 0.838 (95% CI, 0.680-0.938), and specificity of 0.849 (95% CI, 0.739-0.925). RMS are difficult to distinguish from other soft tissue tumors by imaging findings due to their lack of specificity (36). Additionally, Franco et al. (37) could not discover any relevance in imaging characteristics at presentation, such as attenuation and heterogeneity, for determining the pathologic subtype of pediatric RMS. In contrast, NB lesions have different original locations, internal calcifications, and encasement of the vessels compared with RMS (14), and the present study also found imaging feature differences in homogeneity and intraspinal tumor extension between the two groups. According to stepwise multivariate logistic regression, the radiological model was created by three factors, and the ROC curve showed that the radiologic model performed better than the clinical model (AUC: 0.915 vs. 0.878). Subsequently, we integrated both clinical and imaging parameters to construct a composite model that exhibited superior diagnostic efficacy (the AUC of 0.962) compared to individual clinical or radiological factors. The calibration curves showed good agreement between the predicted values and the actual results, and the decision curves showed that the integrated nomogram model had a higher net benefit than the clinical or radiological model alone.

During the study, three radiologists with varying levels of professional expertise conducted independent diagnoses of the patients. The results indicated that the diagnostic effectiveness of senior radiologists may be superior to that of middle-aged and junior radiologists (all *P*<0.05). Moreover, through the comparison to the integrated nomogram model, we found that the radiologist’s diagnosis was significantly lower than those of the integrated nomogram model (*P*<0.001). This demonstrates that the model improves the diagnostic efficiency of between RMS and NB in children, which would have the potential advantage to eliminate the need for invasive biopsies in risky anatomical sites and serve as a tool for making management decisions about treatment.

Some limitations of the present study should be noted. First, it is a retrospective study from a single center with inherited selection bias. Second, the clinical and imaging information of RMS and NB patients was acquired over 10 years (2012 to 2023), which may affect the data extracted. Third, due to the low incidence of the two tumors, the number of patients eligible for enrollment and validating the integrated nomogram model was limited. Future trials are needed to provide more robust evidence and improve the generalizability of the findings.

In conclusion, compared with the clinical model, radiological model, and subjective diagnosis, the integrated nomogram model, with integrated clinical and image features, achieved the best diagnostic value, which could help radiologists differentiate pediatric soft tissue RMS and NB preoperatively, reduce unnecessary interventions, and improve prognosis.

## Data availability statement

The raw data supporting the conclusions of this article will be made available by the authors, without undue reservation.

## Ethics statement

The studies involving humans were approved by the Ethical Committee of the Children’s Hospital of Shanghai/Shanghai Children’s Hospital, Shanghai Jiao tong University (Approval number: 2023R073-E01). The studies were conducted in accordance with the local legislation and institutional requirements. The ethics committee/institutional review board waived the requirement of written informed consent for participation from the participants or the participants’ legal guardians/next of kin because this is a retrospective study, and the study poses minimal risk or harm to the participants, involves no collection of personally identifiable information, and can be adequately protected.

## Author contributions

JS: Formal Analysis, Software, Writing – original draft, Data curation, Investigation. TL: Writing – review & editing, Funding acquisition, Data curation, Supervision. HX: Data curation, Writing – review & editing. RX: Data curation, Writing – review & editing. XC: Data curation, Writing – review & editing. HZ: Data curation, Writing – review & editing. QJ: Formal Analysis, Writing – review & editing. XD: Data curation, Writing – review & editing. WX: Data curation, Writing – review & editing. XY: Conceptualization, Methodology, Writing – review & editing, Supervision.
